# Cytotoxicity and Pharmacogenomics of Medicinal Plants from Traditional Korean Medicine

**DOI:** 10.1155/2013/341724

**Published:** 2013-07-11

**Authors:** Victor Kuete, Ean-Jeong Seo, Benjamin Krusche, Mira Oswald, Benjamin Wiench, Sven Schröder, Henry Johannes Greten, Ik-Soo Lee, Thomas Efferth

**Affiliations:** ^1^Department of Biochemistry, Faculty of Science, University of Dschang, Cameroon; ^2^Department of Pharmaceutical Biology, Institute of Pharmacy and Biochemistry, Johannes Gutenberg University, Staudinger Weg 5, 55128 Mainz, Germany; ^3^HanseMerkur Center for Traditional Chinese Medicine, University Hospital Eppendorf, Hamburg, Germany; ^4^Biomedical Sciences Institute Abel Salazar, University of Porto, Portugal; ^5^Heidelberg School of Chinese Medicine, Heidelberg, Germany; ^6^College of Pharmacy, Chonnam National University, Gwangju, Republic of Korea

## Abstract

*Aim*. The present study was designed to investigate the cytotoxicity of a panel of 280 Korean medicinal plants belonging to 73 families and 198 species against human CCRF-CEM leukemia cells. Selected phytochemicals were investigated in more detail for their mode of action. *Methods*. The resazurin assay was used to determine cytotoxicity of the plant extracts. Microarray-based mRNA expression profiling, COMPARE, and hierarchical cluster analyses were applied to identify which genes correlate with sensitivity or resistance to selected phytochemicals of the Korean plants. *Results*. The results of the resazurin assay showed that cytotoxicity extracts tested at 10 **μ**g/mL from 13 samples inhibited proliferation more than 50% (IC_50_ < 10 **μ**g/mL) and the most active plants are *Sedum middendorffianum* (15.33%) *and Lycoris radiata* (17.61%). Out of 13 selected phytochemicals from these plants, hopeaphenol and deoxynarciclasine were the most cytotoxic ones. Genes from various functional groups (transcriptional or translational regulation, signal transduction, cellular proliferation, intracellular trafficking, RNA metabolism, endoplasmic/sarcoplasmic reticulum function, etc.) were significantly correlated with response of tumor cell lines to these two compounds. *Conclusion*. The results provide evidence on the possible use of selected Korean medicinal plants and chemical constituents derived from them for the treatment of tumors.

## 1. Introduction

Since ancient times, humans have derived many benefits from medicinal plants. A variety of different medicinal plants have traditionally been used in Asian cultures as medicinal plants to treat cancers [1]. The pharmacological screening of plants is an important mean for the discovery of new, safe, and effective drugs. Over 50,000 plants, possess therapeutic virtues in the world, and about 80% of human use herbal medicines at least once in their life [2, 3]. Medicinal plants through the multiplicities of their chemical constituents are important for the discovery of new substances active against tumors and other cancers. Screenings of medicinal plants used as anticancer drugs have provided modern medicine with effective cytotoxic pharmaceuticals. More than 60% of the approved anticancer drugs in the United State of America (from 1983 to 1994) were from natural origin [4, 5]. 

Traditional Korean medicine is widely used in Korea and is the primary health care system for more than 20% of the population [6, 7]. As previously emphasized in a regional demographic survey, about 30–40% of the Korean population had used complementary and alternative medicine (including traditional Korean medicine) within a 5-year period [6, 8]. 

In the pharmacopoeia of many countries worldwide including Korea, there is still a serious lack of information on the use of medicinal plants in the treatment of cancer. However, it has been recommended that ethnopharmacological usages such as for immune and skin disorders, inflammatory, infectious, parasitic, and viral diseases should be taken into account when selecting plants used to treat cancer, since these reflect disease states bearing relevance to cancer or cancer-like symptoms [9, 10]. In general, leukemia cells are often more sensitive to cytotoxic drugs than adherent cancer cells, making them a suitable tool for primary bioactivity screenings. Hence, the present work was designed to investigate the cytotoxicity of a panel of 280 Korean medicinal plants against human CCRF-CEM leukemia cells. Based on the bioactivity of the extracts, selected phytochemicals of active plants were analyzed in more detail. Genes determining sensitivity or resistance to selected compounds were identified by microarray-based mRNA expression profiles and hierarchical cluster analyses in a panel of tumor cell lines of the National Cancer Institute, USA.

## 2. Material and Methods

### 2.1. Plant Material and Extraction

Medicinal plants used in the present work were collected at different localities of the Republic of Korea and provided by Prof. Ik-Soo Lee (College of Pharmacy, Chonnam National University, Kwangju, South Korea). The plants were identified at the National herbarium, where voucher specimens were deposited under the references numbers (see Supplementary material available online at http://dx.doi.org/10.1155/2013/341724). The extraction of the air-dried and powdered plant material was conducted using methanol (HPLC grade) with either ASE 300 (Dionex) or a sonicator (Branson Ultrasonics) at 50°C. The extracts were then conserved at 4°C until further use.

### 2.2. Cell Culture

Human CCRF-CEM leukemia cells were obtained from Professor Axel Sauerbrey (University of Jena, Jena, Germany). Cells were maintained in RPMI 1640 containing 100 units/mL penicillin and 100 mg/mL streptomycin and supplemented with heat-inactivated 10% fetal bovine serum (FBS), in a humidified environment at 37°C with 5% CO_2_. Doxorubicin ≥ 98.0% (Sigma-Aldrich, Schnelldorf, Germany) was used as a positive (cytotoxic) control. 

### 2.3. Resazurin Cell Growth Inhibition Assay

Alamar Blue or Resazurin (Promega, Mannheim, Germany) reduction assay [11] was used to assess the cytotoxicity of the studied samples. The assay tests cellular viability and mitochondrial function. Briefly, aliquots of 5 × 10^4^ cells/mL were seeded in 96-well plates, and extracts were added immediately. After 24 h incubation, 20 *μ*L resazurin 0.01% w/v solution was added to each well and the plates were incubated at 37°C for 1-2 h. Fluorescence was measured on an automated 96-well Infinite M2000 Pro plate reader (Tecan, Crailsheim, Germany) using an excitation wavelength of 544 nm and an emission wavelength of 590 nm. Doxorubicin was used as positive control. The concentration of DMSO was kept at or below 0.1% in all experiments. Each assay was done at least three times, with three replicates each. All samples were tested at a single concentration of 10 *μ*g/mL.

### 2.4. COMPARE and Hierarchical Cluster Analysis of mRNA Microarray Data

The mRNA microarray hybridization of the NCI cell line panel has been described [12, 13] and the date has been deposited at the NCI website (http://dtp.nci.nih.gov/). For hierarchical cluster analysis, objects were classified by calculation of distances according to the closeness of between-individual distances by means of hierarchical cluster analysis. All objects were assembled into a cluster tree (dendrogram). The merging of objects with similar features leads to the formation of a cluster, where the length of the branch indicates the degree of relation. The distance of a subordinate cluster to a superior cluster represents a criterion for the closeness of clusters as well as for the affiliation of single objects to clusters. Thus, objects with tightly related features appear together, while the separation in the cluster tree increases with progressive dissimilarity. Previously, cluster models have been validated for gene expression profiling and for approaching molecular pharmacology of cancer [12–14]. Hierarchical cluster analyses applying the WARD method were done with the WinSTAT program (Kalmia, Cambridge, MA, USA). Missing values were automatically omitted by the program, and the closeness of two joined objects was calculated by the number of data points they contained. In order to calculate distances between all variables included in the analysis, the program automatically standardizes the variables by transforming the data with a mean = 0 and a variance = 1. For COMPARE analysis, the mRNA expression values of genes of interest and IC_50_ values for selected phytochemicals of the NCI cell lines were selected from the NCI database (http://dtp.nci.nih.gov/). The mRNA expression has been determined by microarray analyses as reported [12]. COMPARE analyses were performed by a web-based algorithm (http://dtp.nci.nih.gov/) to produce rank-ordered lists of genes expressed in the NCI cell lines. The methodology has been described previously in detail [15]. Briefly, every gene of the NCI microarray database was ranked for similarity of its mRNA expression to the IC_50_ values for the corresponding compound. Scale indices of correlations coefficients (*R*-values) were created. Pearson's correlation coefficients with positive algebraic signs indicate that greater mRNA expression in cell lines correlate with enhanced drug resistance, whereas coefficients with negative algebraic signs indicate that greater mRNA expression in cell lines was associated with drug sensitivity. Pearson's correlation test and *χ* ^2^ test were implemented into the WinSTAT Program (Kalmia). The one-way ANOVA at 95% confidence level was used for statistical analysis.

## 3. Results 

### 3.1. Cytotoxic Activity

In the present work, we screened 280 plant extracts derived from traditional Korean medicine belonging to 73 plant families and 198 species (see Supplementary Table) for their cytotoxicity against human CCRF-CEM leukemia cells. 

**Figure 1:**
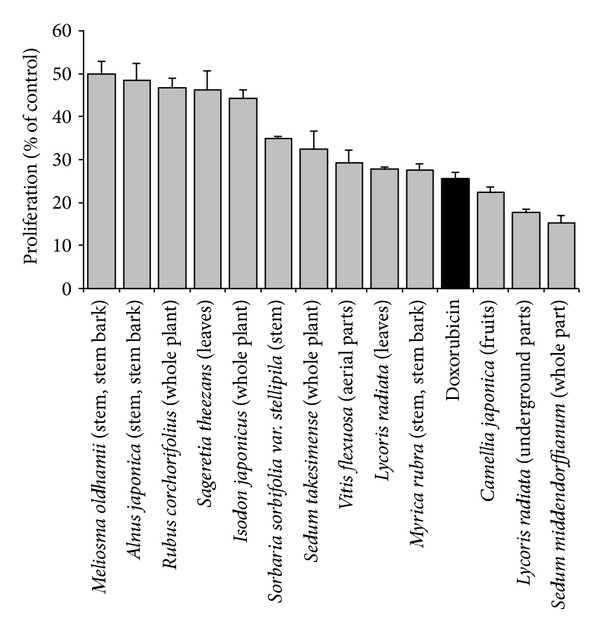
Cytotoxicity of the 13 most active samples from selected Korean medicinal plants extract (at 10 *μ*g/mL) and doxorubicin (1 *μ*M) on CCRF-CEM leukemia cells. Data with different superscript letters are significantly different (*P* < 0.05). (See Supplementary Data for the overview of the cytotoxicity of all 280 tested extracts. Are shown mean values ± SD of each five measurements.) The established anticancer drug, doxorubicin was used as positive control (black bar).

The results of the cytotoxicity assay (Figure 1; Supplementary Table) indicated that is among the 280 studied plant extracts, 58 promoted the growth (% growth > 100%) of CCRF-CEM cells, while the remaining samples showed various extents of growth inhibition. Out of these extracts, 207 extracts showed no or a weak inhibition of cancer cell proliferation (<50%) at the tested concentration of 10 *μ*g/mL. However, 13 samples induced the proliferation of less 50% cells (IC_50_ < 10 *μ*g/mL). These samples included *Sedum middendorffianum *whole plant (15.33%), *Lycoris radiata *underground parts (17.61%), *Camellia japonica *fruits (22.28%), *Myrica rubra *stem-stem bark (27.61%), *Lycoris radiata *leaves (27.73%), *Vitis flexuosa aerial parts *(29.32%), *Sedum takesimense *whole plant (32.39%), *Sorbaria sorbifolia var. stellipila *stems (34.98%), *Isodon japonicus *whole plant (44.15%), *Sageretia theezans *leaves and stems (46.35%), *Rubus corchorifolius* aerial part (46.81%), *Alnus japonica *stem-stem bark (48.43%), and *Meliosma oldhamii *stem-stem bark (49.94%) (Figure 1). The traditional used and reported bioactivities are compiled in Table 1. 

### 3.2. Cytotoxicity of Phytochemicals Derived from Koran Medicinal Plants

**Figure 2:**
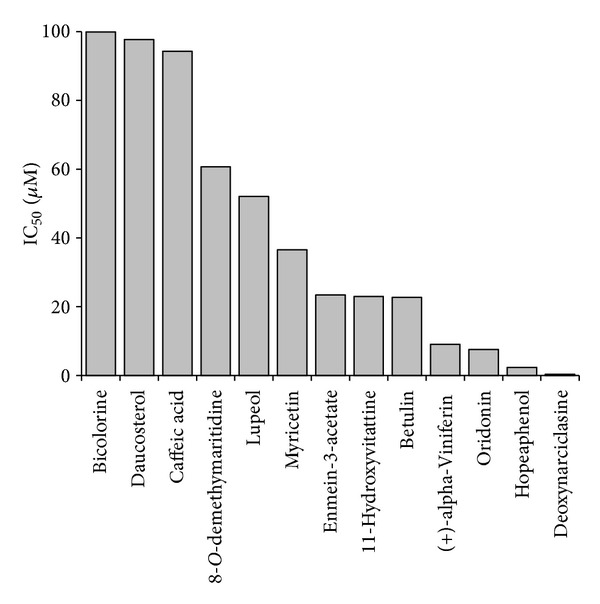
Mean log_10_IC_50_ values for selected phytochemicals derived from Korean medicinal plants for tumor cell lines from the NCI cell line panel.

We searched the literature on the chemical constituents of the cytotoxic Korean plants (Table 1). Subsequently, we mined the NCI database for these compounds. Thirteen compounds with average log_10_IC_50_ values over the entire NCI cell line panel below −4.0 M are depicted in Figure 2. Hopeaphenol and deoxynarciclasine were the most cytotoxic compounds with log_10_IC_50_ values of 0.346 × 10^−6^ M (=0.346 *μ*M) and 0.233 × 10^−5^ M (=2.33 *μ*M), respectively. 

**Figure 3:**
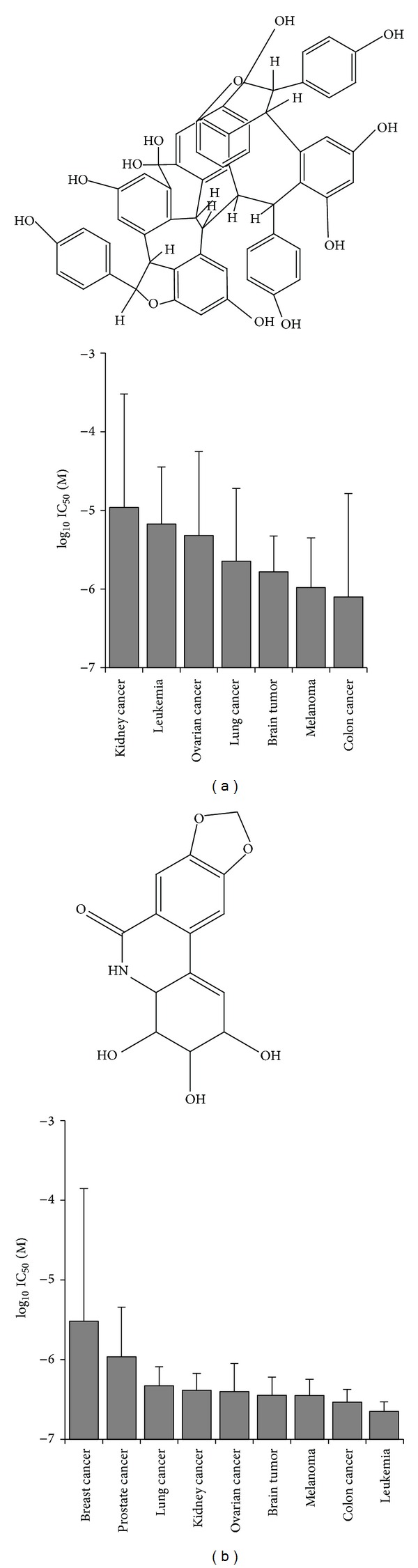
Cytotoxic activity of hopeaphenol (a) and deoxynarciclasine (b) towards cell lines of different tumor types (mean ± SD).

If the average IC_50_ values over the entire range of cell lines were diversified regarding their tumor types, colon cancer and melanoma cell lines were most sensitive towards hopeaphenol, whereas leukemia and kidney cancer cell lines were most resistant (Figure 3(a)). The cell lines reacted in a different manner towards deoxynarciclasine. Leukemia and colon cancer were most sensitive towards this compound, whereas breast cancer and prostate cancer cell lines were most resistant (Figure 3(b)). 

### 3.3. Cross-Resistance of the NCI Cell Line Panel towards Hopeaphenol and Deoxynarciclasine

 The log_10_IC_50_ values of hopeaphenol and deoxynarciclasine were correlated with clinically well-established anticancer drugs. The cell line panel showed statistically significant correlations between deoxynarciclasine and doxorubicin, daunorubicin, vincristine, paclitaxel, cisplatin, melphalan and carmustin. By contrast, cross-resistance was not found between hopeaphenol and these standard drugs, indicating that hopeaphenol might be useful for the treatment of otherwise drug-resistant tumors (Table 2). Interestingly, an inverse correlation was found between the log_10_IC_50_ values for hopeaphenol and the epidermal growth factor receptor tyrosine kinase inhibitor, erlotinib. Such a relationship was not found between deoxynarciclasine and erlotinib (Table 2). 

### 3.4. COMPARE and Hierarchical Cluster Analyses of mRNA Microarray Data

We further investigated the microarray-based transcriptomic mRNA expression by COMPARE analyses to test whether the responses of the tumor cells lines to hopeaphenol and deoxynarciclasine were associated with specific gene expression profiles. For this reason, we mined the transcriptome-wide mRNA expression database of the NCI and correlated the expression data with the IC_50_ values for both compounds. This represents a hypothesis-generating bioinformatical approach, which allows the identification of novel putative molecular determinants of cellular response for hopeaphenol and deoxynarciclasine. The scale rankings of genes obtained by COMPARE computation were subjected to Pearson's rank correlation tests. The thresholds for correlation coefficients were *R* > 0.50 for direct correlations and *R* < −0.50 for inverse correlations. The genes fulfilling these criteria are shown in Table 3 (for hopeaphenol) and Table 4 (for deoxynarciclasine). These genes were from diverse functional groups for hopeaphenol (Table 3). For deoxynarciclasine, genes were found involved in transcription (*ILF2, BCLAF1, MATR3, PSPC1, ZBTB11, CNOT8, IKZF5, SF1, EBP*, and *MYBL1*), RNA metabolism (*HNRNPA1, LARS, SFRS1, FARSA, NUDT21, DDX39, *and *SERBP1*), translation (*GOT1, NGDN*), cellular proliferation (*CWF19L1, MKI67, HSPA9, MLF1IP*, and *DCBLD2*), intracellular trafficking (*OPTN, SNX6*, and *RAB11FIP5*), endoplasmic/sarcoplasmic reticulum function (*SLN, DNAJC10, LMAN2L, SEC24D*, and *KDELR2*), signal transduction (*FRAT2, CHUK, GNA12*, and *LPP*), and others. 

**Figure 4:**
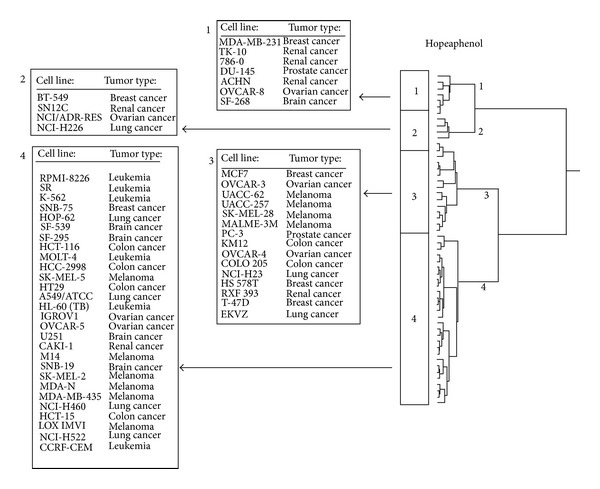
Dendrograms obtained by hierarchical cluster analysis of microarray-based gene expressions for hopeaphenol of the panel of NCI cell lines. The dendrograms were obtained by clustering using the WARD method.

**Figure 5:**
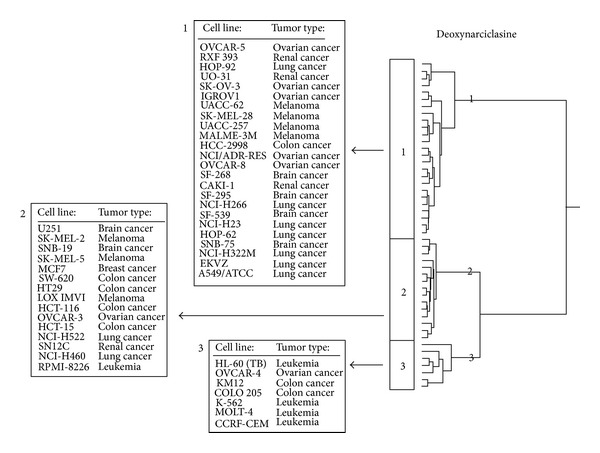
Dendrograms obtained by hierarchical cluster analysis of microarray-based gene expressions for deoxynarciclasine of the panel of NCI cell lines. The dendrograms were obtained by clustering using the WARD method.

As a next step, the genes identified by COMPARE analyses and Pearson's rank correlation tests were subjected to hierarchical cluster analyses. Only the mRNA expression levels but not the IC_50_ values of the compounds for the cell line panel were used for cluster analyses. Four cluster branches were found in the hopeaphenol-related dendrogram (Figure 4) and three clusters were obtained in the deoxynarcilasine-related cluster analysis (Figure 5). Remarkably, the distribution of cell lines sensitive or resistance to both drugs considerably varied between the different clusters of the dendrograms. Since the IC_50_ values of the compounds were not included into the cluster analysis, we could address the question whether the gene expression profiles alone are sufficient to predict sensitivity or resistance of cell lines to the compounds. 

As shown in Table 5, the distributions of cell lines sensitive or resistant to hopeaphenol and deoxynarciclasine, respectively, were significantly different between the clusters of the dendrograms, indicating that the expression of these genes was not only responsible for the branching of the dendrograms, but also predicted sensitivity or resistance of these cell lines towards these compounds. 

## 4. Discussion

### 4.1. Cytotoxic Activity

The cytotoxicity observed in *Isodon. japonicus *in this work is in concordance with previous reports. In effect, the cytotoxic effect of *I. japonicus* extract was reported against five human cancer cell lines (IC_50_ values below 10 *μ*g/mL on stomach MKN-45, breast MCF-7, leukemia K562, colon HT29, and lung A549 cell lines) with leukemia K562 (IC_50_ : 2.70 *μ*g/mL) being the most sensitive [16]. Also, the ethanol extract of *Lycors radiata* exhibited significant antiproliferative effect against B16F10 melanoma cells and induced apoptosis through the activation of p38 and AP-1 pathway [17]. The present report, therefore, provides evidence on the activity of *L. radiata* not only against cell lines derived from solid tumors but also derived from the hematopoietic system, that is, CCRF-CEM leukemia cells. *Sorbaria sorbifolia* was cytotoxic towards HepG-2 cells via induction of apoptosis and cell cycle arrest [18], and evidence of this activity towards CCRF-CEM leukemia cells is provided herein.

In the US NCI plant screening program, a crude extract is generally considered to have *in vitro* cytotoxic activity, if the IC_50_ value following incubation between 48 and 72 h is less than 20 *μ*g/mL [19]. In this study, we reduced the cutoff point to 10 *μ*g/mL. All extracts were tested at this concentration and only samples with an inhibitory effect >50% were considered to have highly promising activities against leukemia cells. Under this condition, 13 samples from 12 medicinal plants (Figure 1) were identified as promising anticancer products and should be screened for more cancer cell lines. It is noteworthy that only *Lycoris radiata *exhibited a significant (<50% growth proliferation) activity with both aerial and subterraneal parts, suggesting that different parts of the plant should be considered when screening the cytotoxicity of medicinal plants. 

To the best of our knowledge, the cytotoxic effect of several active plants (Table 1) against leukemia cells was reported here for the first time. Nevertheless, some of the analyzed plants contain compounds with known cytotoxicity against cancer cells. In fact, the diarylheptanoids [1,7-bis-(3,4-dihydroxyphenyl)-heptane-3-*O-*β*-* d-glucopyranosyl(1→3)-**β**-d-xylopyranoside; 1,7-bis-(3,4-dihydroxyphenyl)-heptane-3-*O-*β*-* d-apiofuranosyl(1→6)-**β**-d-glucopyranoside; 1,7-bis-(3,4-dihydroxyphenyl)-heptane-5-*O-*β*-* d-glucopyranoside, 1,7-bis-(3,4-dihydroxyphenyl)-5-hydroxyheptane; 1,7-bis-(3,4-dihydroxyphenyl)-heptane-3-one-5-*O*-*β*-d-glucopyranoside; oregonin; hirsutanonol; hirsutenone; 1,7-bis-(3,4-dihydroxyphenyl)-5-hydroxyheptane-3-*O-*β*-* d-xylopyranoside and platyphylloside], isolated from the bark of *A. japonica*, showed cytotoxic activities on human, B16 mouse melanoma cells and SNU-1 gastric cancer cell lines with IC_50_ values varying from 17.02 to 55.47 *μ*M [20]. Interestingly, the extract of the stem bark of this plant exhibited significant antiproliferative activity on leukemia CCRF-CEM cells (Figure 1), inducing less than 25% growth at 10 *μ*g/mL. In addition, Kim et al. [21] reported the presence of two well-known cytotoxic compounds, betulin and lupeol [22] in the extract of this plant. Apoptosis induction *via* Fas-mediated pathway was also reported in human MCF-7 breast adenocarcinoma cells by prodelphinidin B-2 3,3′-di-*O*-gallate, a constituent of *M. rubra* [23]. The presence of such cytotoxic compounds could explain the good activity of the crude extract of *A. japonica* and *M. rubra.* Similarly, the presence of cytotoxic compounds such as quercetin or ferulic acid [22], in *R. corchorifolius* and* S. middendorffianum *extracts [24, 25] and *Sedum takesimense *[26] could also provide some explanation on their antiproliferative potentials. Nonetheless, it is interesting to know that the activity does not only depend on the presence of a cytotoxic substance in a plant, but also their quantities and possible interaction with other plant constituents. For example, cytotoxic compounds such as incanone {known to be active on HL60 leukemia cells (IC_50_ value of 6 **µ**M) [25])} or verbascoside [27] {active on human HEP-2 larynx epidermoid carcinoma, human RD rhabdomyosarcoma and human MCF-7 breast adenocarcinoma cell lines [28]} were reported in *Caryopteris incana. *But extract from *Caryopteris incana* did not show significant activities against CCRF-CEM leukemia cells in the present study. 

We screened the NCI database of the Developmental Therapeutics Program of the NCI for the constituents of our panel of Korean medicinal plants. The two most cytotoxic compounds were hopeaphenol and deoxynarciclasine. Both compounds have been previously reported to be cytotoxic [29–32]. While hopeaphenol's activity has been demonstrated in mouse tumors *in vitro* and *in vivo* [31, 32], the present investigation shows that this compounds also active against human tumor cells. To the best of our knowledge, the mechanisms of action of these two compounds have not been investigated in the past and are addressed in our analysis for the first time. 

### 4.2. COMPARE and Hierarchical Cluster Analyses of mRNA Microarray Data

To gain insight into possible modes of action of both phytochemicals, we investigated gene expression profiles of the NCI cell line panel. By microarray-based gene expression and COMPARE analyses, we correlated the IC_50_ values for both compounds of 60 tumor cell lines with transcriptomic mRNA expression levels of this cell line panel [12]. This approach has been successfully used to unravel the mode of action of novel compounds [33]. Cluster and COMPARE analyses are also useful for comparing gene expression profiles with IC_50_ values for investigational drugs to identify candidate genes for drug resistance [34, 35] and to identify prognostic expression profiles in clinical oncology [36, 37].

Eleven genes passing the correlation thresholds of *R* > 0.5 and *R* < −0.5 were identified to significantly correlate with sensitivity or resistance to hopeaphenol. Except for fibroblast growth factor 9 [38], none of them have been associated with response of tumor cells to cytostatic drugs. This point of view is supported by the fact that the NCI cell lines did not exert cross-resistance between hopeaphenol and anticancer drugs, such as doxorubicin, vincristine, cisplatin, and others. 

The genes correlating with the response of tumor cells to deoxynarciclasine were also not known to confer drug resistance as of yet, but many of them belong to functional groups, which are associated with response to chemotherapy. For example, transcriptional regulation, signal transduction, and cell proliferation are well-known processes influencing the success of cancer chemotherapy [39–42]. This might also explain why the NCI cell line panel exhibited cross-resistance between deoxynarciclasine and standard chemotherapy. On the other hand, genes involved in cellular trafficking or endoplasmic/sarcoplasmic reticulum functions have not been recognized as possible mechanisms of drug resistance of tumors. This finding merits more detailed investigations in the future. 

### 4.3. Conclusion

The present work provides evidence that some plants derived from Korean medicine could be useful in the treatment of leukemia and supports the advanced investigation of the most active plants extracts. It also provides first pharmacological data on the cytotoxicity of some plants, such as *Adenophora racemosa, Cinnamomum japonicum, Eurya japonica, Sedum middendorffianum,* and *Vitis flexuosa*. The identification of cytotoxic phytochemicals from these plants, for example, hopeaphenol, and deoxynarciclasine, and the investigation of their possible molecular modes of action may foster the development of novel treatment strategies against otherwise drug-resistant and refractory tumors. 

## Supplementary Material

Extracts of medicinal plants used in traditional Korean medicine investigated for cytotoxic activity towards cancer cells.

## Figures and Tables

**Table 1:** Korean plants with cytotoxic activity.

Plants (and family)	Traditional uses	Part used	Previously reported activity of the plant	Reported chemical constituents
*Alnus japonica* Steudel (Betulaceae)	In oriental traditional medicine as remedies for fever, hemorrhage, diarrhea, and alcoholism [43]	Stems-stem bark	Hepatoprotective and antioxidant activities [44], antiviral activity against the influenza virus [45]	1,7-Bis-(3,4-dihydroxyphenyl)-3-hydroxyheptane-5-*O*-**β**-D-xylopyranoside; 1,7-bis-(3,4-dihydroxyphenyl)-heptane-3-*O*-*β*-D-glucopyranosyl(1→3)-**β**-D-xylopyranoside; 1,7-bis-(3,4-dihydroxyphenyl)-heptane-3-*O*-**β**-D-apiofuranosyl(1→6)-*β*-D-glucopyranoside; 1,7-bis-(3,4-dihydroxyphenyl)-heptane-5-*O*-**β**-D-glucopyranoside; 1,7-bis-(3,4-dihydroxyphenyl)-5-hydroxylheptane; 1,7-bis-(3,4-dihydroxyphenyl)-heptane-3-one-5-*O*-**β**-D-glucopyranoside; oregonin; hirsutanonol; hirsutenone; platyphylloside [44]; tannins (alnusjaponins A and B); 5-*O*-galloyl-(−)-shikimic acid, 2,3-(*S*)-hexahydroxydiphenoyl-D-glucose, 4,6-di-*O*-galloyl-D-glucose, 1,4-di-*O*-galloyl-*β*-D-glucose, 4,6-(*S*)-valoneoyl-D-glucose; strictinin; gemin D; pedunculagin; praecoxin A; flosin A; stachyurin; casuarinin [46], lupeol; betulin; betulinic aldehyde; 3-acetoxybetulinic aldehyde, **β**-sitosterol [45]

*Camellia japonica * L. (Theaceae)	Cosmetic protectant to keep the skin and hair healthy and as a soothing agent [47]	Fruits	Antibacterial activity [48], inhibitor of human immunodeficiency virus type 1 protease [49], Epstein-Barr virus inhibitor [50], antimetastasis activity [51], antioxidant activity [52, 53], inhibitor of human type I procollagen production [54], and antiallergic responses [55], anti-inflammatory [47]	3**β**,18**β**-dihydroxy-28-norolean-12-en-16-one; 18**β**-hydroxy-28-norolean-12-ene-3,16-dione; camelliagenin A, B, and C [56], camellenodiol 3-*O*-**β**-D-galactopyranosyl(1→2)[**β**-D-xylopyranosyl(1→2)-*β*-D-galactopyranosyl(1→3)]-*β*-D-glucuronopyranoside; camellenodiol 3-*O*-4′′-*O*-acetyl-**β**-D-galactopyranosyl(1→2)[**β**-D-xylopyranosyl(1→2)-**β**-D-galactopyranosyl(1→3)]-**β**-D-glucuronopyranoside; camellenodiol 3-*O*-(**β**-D-galactopyranosyl(1→2)[**β**-D-xylopyranosyl(1→2)-**β**-D-galactopyranosyl(1→3)]-6′-methoxy-**β**-D-glucuronopyranoside; maragenin II 3-*O*-(**β**-D-galactopyranosyl(1→2)[**β**-D-xylopyranosyl(1→2)-*β*-D-galactopyranosyl(1→3)]-6′-methoxy-**β**-D-glucuronopyranoside, camellioside A; camellioside B [57]

*Isodon japonicus* (Burman f.) H. Hara (Labiatae)	Antibacterial, anti-inflammation, and anthelmintic [58]	Whole plant	Cytotoxicity on K562 human leukemia cells and immunomodulatory activity [16], antibacterial activity for plant constituents [59]	Isadonol; epinodosin; sodoponin; epinodosinol [60, 61]; epinodosin; oridonin; taihangjaponicain A; lushanrubescensin J; bisjaponins A and B [58]; isodonal, trichodonin; nodosin; enmein; oridonin; enmein-3-acetate [59]

*Lycoris radiata* (L'Her.) Herbert (Amaryllidaceae)	Laryngeal trouble, furuncle, carbuncle, suppurative wounds [62]	Leaves, underground parts	Cytotoxicity against B16F10 melanoma cells [17]	Different types of alkaloids (crinine-type; galanthamine-type; lycorine-type homolycorine-type; tazettine-type; narciclasine-type; and lycorine-type alkaloids); trisphaeridine; galanthine; bicolorine; 11-hydroxyvittatine; 8-*O*-demethymaritidine;*O*-demethylgalanthamine; *O*-demethyllycoramine [63]

*Meliosma oldhamii* Miq. ex. Maxim. (Sabiaceae)	Liver ailments [64]	Stems-stem bark	Low Cholinesterase inhibition (12–19% at 5 mg/mL) [65], moderate alpha glucosidase activity [64]	—

*Myrica rubra* Sieb. and Zucc. (Myricaceae)	Diarrhea; gastroenteritis in China [66]	Stems-stem bark	Antioxidant activity [67]; anti-influenza virus activity [68]	Taraxerone; taraxerol; myricadiol; sitosterol; 28-hydroxy-D-friedoolean-14-en-3-one (); myricanol 5-*O*-*β*-D-glucopyranosyl-(1→3)-*β*-D-glucopyranoside; myricanol 5-*O*-*α*-L-arabinofuranosyl-(1→6)-**β**-D-glucopyranoside; isomyricanone [69]; cyanidin-3-*O*-glucoside; myricetin; quercetin-3-*O*-rutinoside [67]

*Rubus corchorifolius* L. f. (Rosaceae)	Stomachache, diarrhea, and dysentery [18]	Whole plant	Antioxidant activity of essential oil [70]	*Ent*-kauran-3**β**, 16**β**, 17, 19-tetrol; *ent*-2-carbonyl-16**β**-hydroxy-kauran-17**β**-D-glucoside [18]; rubusin A; quercetin; kaempferol [25]

*Sageretia theezans* (L.) Brongn (Rhamnaceae)	Tea materials [71]	Leaves, Stems	Antioxidant activity [72]	7-*O*-methylmearnsitrin; myricetrin, kaempferol 3-*O*-**α**-L-rhamnopyranoside, europetin 3-*O*-**α**-L-rhamnoside, and 7-*O*-methyl quercetin 3-*O*-alpha-L-rhamnopyranoside; 7-*O*-methylmearnsetin 3-*O*-rhamnoside [71, 72]

*Sedum middendorffianum *Maxim. (Crassulaceae)	—	Whole plant	—	kaempferol; quercetin; myricetin; arbutin [24]

*Sedum takesimense* Nakai (Crassulaceae)	—	Whole plant	Antioxidant activities [26]	Ferulic acid; caffeic acid; gallic acid; methyl gallate; myricetin; quercetin; luteolin; rhodalin; rhodalidin; luteolin-7-*O*-**β**-D-glucoside; arbutin; 1-(4-hydroxyphenyl)-2-(3,5-dihydroxyphenyl)-2-hydroxyethanone; gossypetin-8-*O*-**β**-D-xylopyranoside; 2,6-di-*O*-galloylarbutin [26]

*Sorbaria sorbifolia* (L.) A. Br. var. *stellipila* MAX. (Rosaceae)	—	Stems	Antioxidant activities, cytotoxicity [73, 74]	Sutherlandin-5-trans-*p*-coumarate; cardiosdiospermin-5-(4-hydroxy) benzoate [75]; noreugenin; wogonin; 5,7,3′,4′-tetrahydroxy-3-methoxyflavone; protocatechuic acid; benzoic acid; emodin; daucosterol [76]; 5,2′,4′-trihydroxy-6,7,5′-trimethoxyflavone; succinic acid; *p*-hydroxybenzoic acid [77]

*Vitis flexuosa* Thunb. (Vitaceae)	—	Aerial parts	—	Flexuosol A; gnetin A; (+)-epsilon-viniferin; vitisin A; hopeaphenol [78]

(—): not reported; the complete list of the tested plants is available in supplementary material.

**Table 2:** Cross-resistance profile of a panel of cell lines towards hopeaphenol and deoxynarciclasine determined by correlating the IC_50_ values by Pearson's correlation test.

	Hopeaphenol	Deoxynarciclasine
Doxorubicin		
*R*-value	<0.30	0.340
*P*-value	>0.05	0.010
Daunorubicin		
*R*-value	<0.30	0.430
*P*-value	>0.05	0.001
Vincristine		
*R*-value	<0.30	0.331
*P*-value	>0.05	0.012
Paclitaxel		
*R*-value	<0.30	0.330
*P*-value	>0.05	0.012
Cisplatin		
*R*-value	<0.30	0.300
*P*-value	>0.05	0.020
Melphalan		
*R*-value	<0.30	0.388
*P*-value	>0.05	0.004
Carmustin		
*R*-value	<0.30	0.394
*P*-value	>0.05	0.003
Erlotinib		
*R*-value	−0.353	>−0.30
*P*-value	0.004	>0.05

Pearson's rank correlation test.

**Table 3:** Genes identified by standard or reverse COMPARE analyses, whose mRNA expression in a panel of 60 cell lines correlated with IC_50_ values for hopeaphenol.

Pearson's correlation coefficient	Experimental ID	Gene symbol	Name	Function
0.552	GC63503	HMGN4	High mobility group nucleosomal binding domain 4	Binds nucleosomal DNA
0.527	GC190712	UAP1	UDP-N-acetylglucosamine pyrophosphorylase 1	Nucleotidyltransferase
0.521	GC45602	TBC1D2	TBC1 domain family, member 2	GTPase activator
0.521	GC188142	ERBB2IP	Erbb2 interacting protein	Receptor adapter, structural constituent of cytoskeleton
0.515	GC26884	PRPS1	Phosphoribosyl pyrophosphate synthetase 1	Involved in nucleotide synthesis
0.513	GC38343	CNNTAL1		Unknown
−0.591	GC97260	MANBA	Mannosidase, beta A, lysosomal	Exoglycosidase
−0.574	GC73531	FGF9	Fibroblast growth factor 9 (glia-activating factor)	Cell growth and differentiation during development
−0.534	GC55495	CYP7B1	Cytochrome P450, family 7, subfamily B, polypeptide 1	Monooxygenase
−0.516	GC94617	GABRA3	Gamma-aminobutyric acid (GABA) A receptor, alpha 3	Neurotransmission
−0.515	GC184017	HES1	Hairy and enhancer of split 1, (Drosophila)	Transcriptional repressor

Information on gene functions was taken from the OMIM database, NCI, USA, (http://www.ncbi.nlm.nih.gov/Omim/) and from the GeneCard database of the Weizmann Institute of Science, Rehovot, Israel (http://bioinfo.weizmann.ac.il/cards/index.html).

**Table 4:** Genes identified by standard or reverse COMPARE analyses, whose mRNA expression in a panel of 60 cell lines correlated with IC_50_ values for deoxynarciclasine.

Pearson's correlation coefficient	Experimental ID	Gene symbol	Name	Function
0.572	GC74997	SNAP25	Synaptosomal-associated protein, 25 kDa	Regulation of neurotransmitter release
0.56	GC32186	OPTN	Optineurin	Maintenance of membrane trafficking
0.557	GC52658	FAM116B	Family with sequence similarity 116, member B	Unknown
0.531	GC187393	ZDHHC7	Zinc finger, DHHCtype containing 7	Palmitoyltransferase
0.529	GC12575	CLEC9A	C-type lectin domain family 9, member A	Endocytic receptor for necrotic cells
0.528	GC188718	SNX6	Sorting nexin 6	Involved in intracellular trafficking
0.528	GC154565	RAB11FIP5	RAB11 family interacting protein 5 (class I)	Involved in protein trafficking
0.527	GC18484	NTAN 1	N-terminal asparagine amidase	Ubiquitin-dependent turnover of intracellular proteins
0.526	GC16433	DNAJC10	DnaJ (Hsp40) homolog, subfamily C, member 10	Endoplasmic reticulum cochaperone
0.524	GC10009	PCOLCE2	Procollagen C-endopeptidase enhancer 2	Binds to procollagens
0.524	GC45803	LMAN2L	Lectin, mannose-binding 2-like	Regulation of export from the endoplasmic reticulum
0.524	GC82947	NGDN	Neuroguidin, EIF4E binding protein	Involved in the translational repression
0.519	GC75800	SEC24D	SEC24 family, member D (*S. cerevisiae*)	Transport of ER-derived vesicles
0.517	GC90440	KDELR2	KDEL (Lys-Asp-Glu-Leu) endoplasmic reticulum protein retention receptor 2	Retention of luminal endoplasmic reticulum proteins
0.514	GC173264	GNA12	Guanine nucleotide binding protein (G protein) *α*12	Transducer in transmembrane signaling
0.512	GC170473	MYBL1	V-Myb myeloblastosis viral oncogene homolog (avian)-like 1	Transcriptional activator
0.512	GC175225	LPP	LIM domain containing preferred translocation partner in lipoma	Signal transduction from cell adhesion sites to the nucleus
0.511	GC14006	ITGB5	Integrin, *β*5	Receptor for fibronectin
0.509	GC150035	DCBLD2	Discoidin, CUB, and LCCL domain containing 2	Involved in tumor progression
0.508	GC73833	NCEH1	Neutral cholesterol ester hydrolase 1	Promotes tumor cell migration
0.507	GC177466	AHNAK	*AHNAK* nucleoprotein	Involved in neuronal cell differentiation
0.502	GC40315	ADAL	Adenosine deaminaselike	Putative nucleoside deaminase
0.502	GC28174	MPG	N-methylpurine-DNA glycosylase	Hydrolysis of alkylated DNA
0.501	GC18079	CR1	Complement component (3b/4b) receptor 1 (Knops blood group)	Mediates cellular binding of particles and immune complexes
−0.658	GC18354	ILF2	Interleukin enhancer binding factor 2, 45 kDa	Transcription
−0.608	GC44240	NDUFA2	NADH dehydrogenase (ubiquinone) 1 alpha subcomplex, 2, 8 kDa	Part of mitochondrial membrane respiratory chain
−0.605	GC65788	HNRNPA1	Heterogeneous nuclear ribonucleoprotein A1	Involved in RNA replication
−0.589	GC43237	CWF19L1	CWF19-like 1, cell cycle control (*S. pombe*)	Cell cycle control
−0.572	GC174320	BCLAF1	BCL2-associated transcription factor 1	Death-promoting transcriptional repressor
−0.572	GC40779	LARS	Leucyl-tRNA synthetase	Editing of tRNA
−0.567	GC151509	FRAT2	Frequently rearranged in advanced T-cell lymphomas 2	Wnt signaling regulator
−0.561	GC175610	IK	*IK* cytokine, downregulator of HLA II	May bind to chromatin
−0.555	GC30213	SLN	Sarcolipin	Sarcoplasmic reticulum proteolipid
−0.553	GC162737	ANKHD2		Unknown
−0.552	GC17532	SFRS28		Unknown
−0.55	GC185046	SFRS1	Serine/arginine-rich splicing factor 1	Splicing regulator
−0.55	GC37292	FARSA	Phenylalanyl-tRNA synthetase, *α* subunit	Phenylalanine-tRNA ligase
−0.548	GC32318	MKI67	Antigen identified by monoclonal antibody Ki-67	Cell proliferation
−0.548	GC149101	MATR3	Matrin 3	Regulator of transcription
−0.547	GC150688	PSPC1	Paraspeckle component 1	Regulator of androgen receptor-mediated gene transcription
−0.546	GC80581	ZBTB11	Zinc finger and BTB domain containing 11	Regulator of transcription
−0.546	GC52470	MLF1IP	MLF1 interacting protein	Involved in mitotic progression
−0.545	GC81490	NUDT21	Nudix (nucleoside diphosphate linked moiety X)-type motif 21	Involved in pre-mRNA 3′-processing
−0.543	GC33504	DDX39	DEAD (Asp-Glu-Ala-Asp) box polypeptide 39A	Involved in pre-mRNA splicing
−0.543	GC35760	NDUGB8		Unknown
−0.539	GC36766	CHUK	Conserved helix-loop-helix ubiquitous kinase	Involved in NF-kappa-B signaling
−0.538	GC33463	POLD1	Polymerase (DNA directed), *δ*1, catalytic subunit	DNA synthesis
−0.537	GC83208	SERBP1	SERPINE1 mRNA binding protein 1	Regulation of mRNA stability
−0.536	GC153558	CNOT8	CCR4-NOT transcription complex, subunit 8	Transcription factor
−0.535	GC17771	GOT1	Glutamic-oxaloacetic transaminase 1, soluble (aspartate aminotransferase 1)	Involved in amino acid metabolism
−0.535	GC43091	NUDCD2	NudC domain containing 2	Unknown
−0.534	GC31949	HSPA9	Heat shock 70 kDa protein 9 (mortalin)	Control of cell proliferation and cellular aging
−0.533	GC91403	IKZF5	IKAROS family zinc finger 5 (Pegasus)	Transcriptional repressor
−0.533	GC31641	SF1	Splicing factor 1	Transcriptional repressor
−0.532	GC161453	KDM2B	Lysine (K)-specific demethylase 2B	Histone demethylase
−0.53	GC35520	EBP	CCAAT/enhancer binding protein (C/*EBP*), gamma	Transcription factor
−0.53	GC10226	ABCB7	ATP-binding cassette, sub-family B (MDR/TAP), member 7	Heme transport

Information on gene functions was taken from the OMIM database, NCI, USA, (http://www.ncbi.nlm.nih.gov/Omim/) and from the GeneCard database of the Weizmann Institute of Science, Rehovot, Israel (http://bioinfo.weizmann.ac.il/cards/index.html).

**Table 5:** Separation of clusters of 60 cancer cell lines obtained by hierarchical cluster analysis for hopeaphenol and deoxynarciclasine. The log_10_ IC_50_ median values (M) of each compound were used as cut-off values to define cell lines as being sensitive or resistant. *P* > 0.05 was considered as not significant (*χ* ^2^ test).

	Sensitive	Resistant	*χ* ^2^ test
Hopeaphenol			
Partition*	<−5.80	>−5.80	
Cluster 1	0	7	
Cluster 2	0	4	
Cluster 3	15	0	
Cluster 4	12	16	*P* = 4.49 × 10^−6^

Deoxynarciclasine			
Partition*	<−6.40	>−6.40	
Cluster 1	3	22	
Cluster 2	13	2	
Cluster 3	7	0	*P* = 1.76 × 10^−6^

*log_10_ IC_50_ (M).
