# Supplementary material for: Cytotoxicity and Pharmacogenomics of Medicinal Plants from Traditional Korean Medicine
Source: Evid Based Complement Alternat Med. 2013 Jul 11;2013:341724. doi: 10.1155/2013/341724 (PMC3725712; doi:10.1155/2013/341724)
Supplement: Supplementary file 1 — Extracts of medicinal plants used in traditional Korean medicine investigated for cytotoxic activity towards cancer cells. [file 341724.f1.pdf]

Cytotoxicity towards cancer cells and pharmacogenomics of medicinal plants from traditional Korean medicine

Victor Kuete*, Ean-Jeong Seo*, Benjamin Krusche, Mira Oswald, Benjamin Wiench, Sven Schröder, Henry Johannes Greten, Ik-Soo Lee, Thomas Efferth

- -

Supplementary Table: Extracts of medicinal plants used in traditional Korean medicine investigated for cytotoxic activity towards cancer cells

Samples names	Part	Identification Codes	Extraction solvent
Equisetum hyemale	Whole plant	PB1136.1	MeOH
Osmunda japonica	Whole plant	PB1149.1	MeOH
Matteuccia struthiopteris	Whole plant	PB1194.1	MeOH
Dryopteris lacera	Whole plant	PB1233.1	MeOH
Torreya nucifera	Leaves	PB1363.1	MeOH
Torreya nucifera	Stems	PB1363.2	MeOH
Abies koreana	Leaves	PB1374.3	MeOH
Abies koreana	Stems	PB1374.4	MeOH
Pinus thunbergii	Stems-Heartwood	PB1401.2	MeOH
Pinus thunbergii	Stems-Stembark	PB1401.3	MeOH
Pinus thunbergii	Leaves	PB1401.4	MeOH
Arisaema ringens	Whole plant	PB1947.1	MeOH
Arisaema amurense var. serratum	Whole plant	PB1951.1	MeOH
Symplocarpus nipponicus	Whole plant	PB1955.1	MeOH
Luzula capitata	Whole plant	PB1982.1	MeOH
Heloniopsis orientalis	Whole plant	PB2011.1	MeOH
Hosta minor	Whole plant	PB2027.1	MeOH
Hemerocallis minor	Underground parts	PB2044.3	MeOH
Allium victorialis var. platyphyllum	Roots	PB2048.1	MeOH
Allium grayi	Whole plant	PB2052.1	MeOH
Majanthemum dilatatum	Whole plant	PB2114.3	MeOH
Paris verticillata	Whole plant	PB2122.2	MeOH
Trillium kamtschaticum	Whole plant	PB2124.1	MeOH
Lycoris radiata	Leaves	PB2148.1	MeOH
Lycoris radiata	Underground parts	PB2148.2	MeOH
Dioscorea batatas	Fruits	PB2154.2	MeOH
Cremastra appendiculata	Whole plant	PB2247.1	MeOH
Piper kadzura	Leaves	PB2263.1	MeOH
Salix hultenii	Flowers	PB2299.2	MeOH
Myrica rubra	Stems-Heartwood	PB2326.2	MeOH
Myrica rubra	Stems-Stembark	PB2326.3	MeOH

Alnus japonica	Leaves	PB2350.1	MeOH
Alnus japonica	Stems-Heartw ood	PB2350.2	MeOH
Alnus japonica	Stems-Stembark	PB2350.3	MeOH
Alnus maximowiczii	Leaves	PB2364.4	MeOH
Carpinus laxiflora	Stems-Heartw ood	PB2373.2	MeOH
Castanopsis cuspidata var. sieboldii	Leaves	PB2392.1	MeOH
Quercus acutissima	Leaves	PB2395.1	MeOH
Quercus acutissima	Stems-Heartw ood	PB2395.2	MeOH
Quercus acutissima	Stems-Stembark	PB2395.3	MeOH
Quercus acuta	Leaves	PB2418.1	MeOH
Quercus acuta	Stems	PB2418.2	MeOH
Quercus glauca	Stems-Heartw ood	PB2420.2	MeOH
Quercus salicina	Leaves	PB2423.1	MeOH
Quercus salicina	Stems-Heartw ood	PB2423.2	MeOH
Quercus salicina	Stems-Stembark	PB2423.3	MeOH
Quercus gilva	Leaves	PB2425.1	MeOH
Quercus gilva	Stems-Heartw ood	PB2425.2	MeOH
Quercus gilva	Stems-Stembark	PB2425.3	MeOH
Celtis choseniana	Fruits	PB2447.3	MeOH
Broussonetia kazinoki var. humilis	Leaves	PB2471.1	MeOH
Broussonetia kazinoki var. humilis	Stems	PB2471.2	MeOH
Ficus erecta	Fruits	PB2475.1	MeOH
Ficus erecta	Stems-Heartw ood	PB2475.3	MeOH
Ficus thunbergii	Leaves	PB2479.1	MeOH
Ficus thunbergii	Stems	PB2479.2	MeOH
Loranthus yadoriki	Leaves	PB2515.1	MeOH
Loranthus yadoriki	Stems	PB2515.2	MeOH
Pseudixus japonicus	Whole plant	PB2519.1	MeOH
Asarum maculatum	Whole plant	PB2522.1	MeOH
Asarum sieboldii	Whole plant	PB2523.1	MeOH
Rumex japonica	Whole plant	PB2529.1	MeOH
Bistorta manshuriensis	Whole plant	PB2541.1	MeOH
Reynoutria sachalinensis	Leaves	PB2552.1	MeOH
Reynoutria sachalinensis	Fruits	PB2552.3	MeOH
Reynoutria sachalinensis	Roots	PB2552.6	MeOH
Phytolacca esculenta	Leaves, Stems	PB2638.1	MeOH
Phytolacca esculenta	Roots	PB2638.2	MeOH
Cercidiphyllum japonicum	Leaves	PB2719.1	MeOH
Cercidiphyllum japonicum	Stems-Heartw ood	PB2719.2	MeOH
Cercidiphyllum japonicum	Stems-Stembark	PB2719.3	MeOH
Anemone koraiensis	Whole plant	PB2759.1	MeOH
Anemone reflexa	Whole plant	PB2761.1	MeOH
Ranunculus japonicus	Whole plant	PB2770.1	MeOH
Aconitum longecassidatum	Whole plant	PB2808.1	MeOH
Caltha palustris var. membranacea	Whole plant	PB2846.1	MeOH
Stauntonia hexaphylla	Leaves	PB2861.1	MeOH
Berberis koreana	Leaves, Stems	PB2863.1	MeOH

Berberis amurensis var. lactifolia	Stems	PB2867.3	MeOH
Epimedium koreanum	Whole plant	PB2873.1	MeOH
Sinomenium acutum	Stems, Roots	PB2878.2	MeOH
Magnolia kobus	Leaves	PB2885.1	MeOH
Magnolia kobus	Stems-Heartw ood	PB2885.2	MeOH
Magnolia kobus	Stems-Stembark	PB2885.3	MeOH
Schisandra chinensis	Leaves, Flow ers	PB2892.1	MeOH
Schisandra chinensis	Stems	PB2892.2	MeOH
Illicium religiosum	Leaves	PB2898.1	MeOH
Lindera glauca	Stems	PB2904.1	MeOH
Cinnamomum camphora	Stems-Heartw ood	PB2906.2	MeOH
Cinnamomum camphora	Stems-Stembark	PB2906.3	MeOH
Cinnamomum japonicum	Leaves	PB2907.4	MeOH
Cinnamomum japonicum	Stems	PB2907.5	MeOH
Machilus thunbergii	Stems-Heartw ood	PB2910.2	MeOH
Machilus japonica	Tw igs	PB2912.2	MeOH
Actinodaphne lancifolia	Leaves	PB2915.1	MeOH
Actinodaphne lancifolia	Tw igs	PB2915.2	MeOH
Litsea japonica	Leaves	PB2916.1	MeOH
Hylomecon vernale	Whole plant	PB2923.1	MeOH
Corydalis grandicalyx	Whole plant	PB2931A.1	MeOH
Corydalis incisa	Whole plant	PB2937.2	MeOH
Corydalis heterocarpa var. aponica	Aerial parts	PB2941.1	MeOH
Lepidium ruderale	Whole plant	PB2955.1	MeOH
Cardamine amaraeformis	Whole plant	PB2963A.1	MeOH
Wasabia koreana	Whole plant	PB2972.1	MeOH
Sedum takesimense	Whole plant	PB3020.3	MeOH
Sedum middendorffianum	Whole plant	PB3029.1	MeOH
Kirengeshoma koreana	Aerial parts	PB3072.1	MeOH
Kirengeshoma koreana	Roots	PB3072.2	MeOH
Ribes fasciculatum var. chinense	Stems	PB3107.2	MeOH
Distylium racemosum	Tw igs	PB3118.2	MeOH
Hamamelis japonica	Flow ers, Stems	PB3121.1	MeOH
Sorbaria sorbifolia var. stellipila	Leaves	PB3128.1	MeOH
Sorbaria sorbifolia var. stellipila	Stems	PB3128.2	MeOH
Spiraea prunifolia var. simpliciflora	Branches, Leaves	PB3132.1	MeOH
Physocarpus intermedius	Leaves, Stems	PB3155.1	MeOH
Aruncus dioicus var. kamtschaticus	Flow ers	PB3161.1	MeOH
Rhodotypos scandens	Fruits	PB3163.1	MeOH
Waldsteinia ternata	Whole plant	PB3193.1	MeOH
Rubus corchorifolius	Aerial parts	PB3199.1	MeOH
Rubus takesimensis	Leaves	PB3203.1	MeOH
Rubus takesimensis	Roots	PB3203.2	MeOH
Rubus takesimensis	Stems	PB3203.3	MeOH
Rubus idaeus var. microphullus	Whole plant	PB3211.1	MeOH
Rubus hirsutus	Whole plant	PB3220.2	MeOH
Rosa multiflora	Branches, Leaves	PB3244.1	MeOH

Prunus buergeriana	Leaves	PB3294.1	MeOH
Prunus buergeriana	Stems-Stembark	PB3294.2	MeOH
Prunus buergeriana	Stems-Heartwood	PB3294.3	MeOH
Prunus yedoensis	Leaves, Flowers	PB3304.1	MeOH
Prunus yedoensis	Stems	PB3304.2	MeOH
Prunus pendula for. ascendens	Leaves	PB3306.1	MeOH
Prunus pendula for. ascendens	Stems-Heartwood	PB3306.2	MeOH
Prunus pendula for. ascendens	Stems-Stembark	PB3306.3	MeOH
Prunus serrulata var. spontanea	Leaves	PB3307.1	MeOH
Prunus serrulata var. spontanea	Stems-Heartwood	PB3307.2	MeOH
Prunus serrulata var. spontanea	Stems-Stembark	PB3307.3	MeOH
Crataegus pinnatifida	Fruits	PB3329.1	MeOH
Eriobotrya japonica	Leaves	PB3339.1	MeOH
Eriobotrya japonica	Stems-Heartwood	PB3339.2	MeOH
Eriobotrya japonica	Stems-Stembark	PB3339.3	MeOH
Chaenomeles lagenaria	Stems	PB3344.1	MeOH
Pyrus ussuriensis	Leaves	PB3355.1	MeOH
Pyrus calleryana var. fauriei	Fruits	PB3367.1	MeOH
Pourthiaea villosa var. brunnea	Stems	PB3374.2	MeOH
Caesalpinia japonica	Leaves, Stems	PB3403.1	MeOH
Maackia fauriei	Leaves	PB3413.1	MeOH
Maackia fauriei	Stems-Heartwood	PB3413.2	MeOH
Maackia fauriei	Stems-Stembark	PB3413.3	MeOH
Lespedeza maximowiczii	Leaves, Stems	PB3421.1	MeOH
Vicia angustifolia var. segetalis	Whole plant	PB3466.3	MeOH
Caragana sinica	Stems	PB3534.1	MeOH
Oxalis articulata	Whole plant	PB3578A.1	MeOH
Phellodendron insulare	Stems-Stembark	PB3598.6	MeOH
Citrus dachibana	Leaves	PB3607A.1	MeOH
Citrus dachibana	Stems-Heartwood	PB3607A.2	MeOH
Citrus dachibana	Stems-Stembark	PB3607A.3	MeOH
Melia azedarach var. japonica	Stems-Heartwood	PB3616.2	MeOH
Sapium japonicum	Stems-Stembark	PB3628.3	MeOH
Euphorbia jolkini	Aerial parts	PB3642.1	MeOH
Ilex crenata var. microphylla	Leaves	PB3662.1	MeOH
Ilex crenata var. microphylla	Stems	PB3662.2	MeOH
Ilex cornuta	Leaves	PB3663.1	MeOH
Ilex cornuta	Stems-Heartwood	PB3663.2	MeOH
Ilex cornuta	Stems-Stembark	PB3663.3	MeOH
Ilex rotunda	Stems	PB3665.2	MeOH
Euonymus alatus	Pericarpium	PB3671.3	MeOH
Euonymus trapococcus	Leaves	PB3680.1	MeOH
Euonymus sieboldiana	Fruits	PB3681.1	MeOH
Acer mono	Leaves	PB3699.1	MeOH
Acer mono	Stems-Heartwood	PB3699.2	MeOH
Acer mono	Stems-Stembark	PB3699.3	MeOH
Acer tschonoskii var. rubripes	Leaves	PB3708.4	MeOH

Acer tschonoskii var. rubripes	Stems	PB3708.5	MeOH
Acer barbinerve	Leaves	PB3709.1	MeOH
Acer micro-sieboldianum	Stems	PB3715.1	MeOH
Acer micro-sieboldianum	Leaves	PB3715.2	MeOH
Acer pseudo-sieboldianum var. koreanum	Leaves, Stems	PB3717.1	MeOH
Acer okamotoanum	Stems	PB3723.2	MeOH
Acer okamotoanum	Leaves	PB3723.6	MeOH
Koelreuteria paniculata	Stems	PB3737.3	MeOH
Meliosma oldhamii	Stems-Stembark	PB3739.2	MeOH
Meliosma oldhamii	Stems-Heartw ood	PB3739.3	MeOH
Sageretia theezans	Fruits	PB3751.2	MeOH
Sageretia theezans	Leaves, Stems	PB3751.3	MeOH
Vitis flexuosa	Aerial parts	PB3771.3	MeOH
Elaeocarpus sylvestris var. ellipticus	Leaves	PB3781.1	MeOH
Elaeocarpus sylvestris var. ellipticus	Stems	PB3781.2	MeOH
Actinidia arguta var. platyphylla	Leaves	PB3818.1	MeOH
Actinidia arguta var. platyphylla	Stems	PB3818.2	MeOH
Stewartia koreana	Leaves	PB3820.1	MeOH
Stewartia koreana	Stems	PB3820.2	MeOH
Camellia japonica	Leaves	PB3823.4	MeOH
Camellia japonica	Stems-Heartw ood	PB3823.5	MeOH
Camellia japonica	Stems-Stembark	PB3823.6	MeOH
Camellia japonica	Fruits	PB3823.7	MeOH
Cleyera japonica	Leaves	PB3827.1	MeOH
Cleyera japonica	Stems-Heartw ood	PB3827.2	MeOH
Cleyera japonica	Stems-Stembark	PB3827.3	MeOH
Eurya japonica	Leaves	PB3828.1	MeOH
Eurya japonica	Stems-Heartw ood	PB3828.2	MeOH
Eurya japonica	Stems-Stembark	PB3828.3	MeOH
Eurya emarginata	Leaves	PB3831.1	MeOH
Eurya emarginata	Stems	PB3831.2	MeOH
Viola albida	Whole plant	PB3848.1	MeOH
Viola keiskei	Whole plant	PB3850.1	MeOH
Viola japonica	Whole plant	PB3864.2	MeOH
Viola tokubuchiana var. takedana	Whole plant	PB3873.1	MeOH
Idesia polycarpa	Leaves	PB3898.1	MeOH
Idesia polycarpa	Stems	PB3898.5	MeOH
Xylosma congestum	Leaves	PB3899.1	MeOH
Xylosma congestum	Stems-Stembark	PB3899.3	MeOH
Elaeagnus umbellata	Leaves, Stems, Flowers	PB3912.2	MeOH
Elaeagnus glabra	Leaves	PB3917.3	MeOH
Elaeagnus glabra	Stems-Heartw ood	PB3917.4	MeOH
Fatsia japonica	Fruits	PB3966.4	MeOH
Acanthopanax sessilifolrus	Stems	PB3973.1	MeOH
Acanthopanax chiisanensis	Leaves	PB3975.1	MeOH
Acanthopanax senticosus	Leaves, Stems	PB3977.1	MeOH
Angelica gigas	Whole plant	PB4038.1	MeOH

Angelica japonica	Leaves	PB4043.1	MeOH
Angelica japonica	Stems, Roots	PB4043.2	MeOH
Ostericum koreanum	Whole plant	PB4048.1	MeOH
Heracleum moellendorffii	Leaves	PB4056.1	MeOH
Heracleum moellendorffii	Underground parts	PB4056.2	MeOH
Aucuba japonica	Stems-Heartw ood	PB4061.5	MeOH
Aucuba japonica	Stems-Stembark	PB4061.6	MeOH
Cornus controversa	Leaves	PB4064.2	MeOH
Cornus controversa	Stems-Stembark	PB4064.3	MeOH
Cornus alba	Leaves, Stems	PB4065.1	MeOH
Rhododendron yedoense var. poukhanense	Leaves, Stems	PB4102.1	MeOH
Vaccinium bracteatum	Leaves	PB4117.1	MeOH
Vaccinium bracteatum	Stems-Stembark	PB4117.3	MeOH
Vaccinium oldhami	Leaves	PB4119.1	MeOH
Vaccinium oldhami	Stems	PB4119.2	MeOH
Ardisia crenata	Leaves	PB4126.1	MeOH
Ardisia crenata	Stems	PB4126.2	MeOH
Primula jesoana	Whole plant	PB4145.1	MeOH
Symplocos chinensis for. pilosa	Stems	PB4161.2	MeOH
Styrax japonica	Leaves	PB4168.5	MeOH
Styrax japonica	Stems-Stembark	PB4168.7	MeOH
Ligustrum japonicum	Leaves	PB4183.1	MeOH
Ligustrum japonicum	Tw igs	PB4183.2	MeOH
Osmanthus insularis	Stems-Heartw ood	PB4199.2	MeOH
Forsythia koreana	Leaves, Stems, Flow ers	PB4206.1	MeOH
Forsythia nakaii	Stems	PB4208.1	MeOH
Syringa velutina var. kamibayashii 'Miss Kim'	Leaves, Stems	PB4223A.1	MeOH
Trigonotis nakaii	Whole plant	PB4315.1	MeOH
Callicarpa japonica var. leucocarpa	Fruits	PB4325.1	MeOH
Caryopteris incana	Leaves, Stems	PB4338.1	MeOH
Ajuga decumbens	Whole plant	PB4341.2	MeOH
Scutellaria indica	Whole plant	PB4353.1	MeOH
Lamium album var. barbatum	Whole plant	PB4385.2	MeOH
Salvia chanroenica	Whole plant	PB4391.1	MeOH
Isodon japonicus	Whole plant	PB4422.1	MeOH
Scrophularia kakudensis	Roots	PB4463.1	MeOH
Scrophularia kakudensis	Aerial parts	PB4463.2	MeOH
Catalpa bignonioides	Stems	PB4541.2	MeOH
Mitchella undulata	Whole plant	PB4583.1	MeOH
Galium spurium	Whole plant	PB4599.1	MeOH
Sambucus sieboldiana	Leaves	PB4622.1	MeOH
Sambucus sieboldiana	Stems-Heartw ood	PB4622.2	MeOH
Sambucus sieboldiana	Stems-Stembark	PB4622.3	MeOH
Weigela florida	Leaves, Flow ers	PB4655.3	MeOH
Weigela florida	Stems	PB4655.4	MeOH
Lonicera chrysantha var. crassipes	Leaves, Flow ers	PB4672.1	MeOH

Lonicera coreana	Leaves	PB4687.1	MeOH
Lonicera coreana	Stems	PB4687.2	MeOH
Lonicera vidalii	Leaves	PB4688.1	MeOH
Trichosanthes kirilowii var. japonica	Fruit flesh	PB4725.2	MeOH
Adenophora racemosa	Whole plant	PB4737A.1	MeOH
Campanula takesimana	Whole plant	PB4744.2	MeOH
Campanula glomerata var. dahurica	Whole plant	PB4747.1	MeOH
Gnaphalium japonicum	Whole plant	PB4766.1	MeOH
Adenocaulon himalaicum	Whole plant	PB4924.1	MeOH
Taraxacum mongolicum	Whole plant	PB5023.2	MeOH
Ixeris stolonifera	Whole plant	PB5036.1	MeOH
Sonchus oleraceus	Whole plant	PB5051.2	MeOH
Youngia japonica	Whole plant	PB5053.2	MeOH
Arisaema takesimense	Fruits	PB5059.4	MeOH
Orostachys iwarenge	Whole plant	PB5060.1	MeOH
